# The Case of the Late William Henry Harrison, President of the United States

**Published:** 1841-05-15

**Authors:** Thomas Miller

**Affiliations:** Washington City


### MEDICAL EXAMINER.

DEVOTED TO MEDICINE, SURGERY, AND THE COLLATERAL SCIENCES.

No. 20.] PHILADELPHIA, SATURDAY, MAY 15, 1841. [Vol. IV.

### ORIGINAL COMMUNICATION.

### THE CASE OF THE LATE WILLIAM HENRY HARRISON, PRESIDENT OF THE UNITED STATES.

*Reported by* Thomas Miller, M. D., &c., *Washington City.*

On the 26th of March, 1841, at 5 o'clock, P. M., I was summoned to visit President Harrison. I found him slightly ailing, although not confined to his room. He complained of having been somewhat indisposed for several days, which he attributed to the great fatigue and mental anxiety he had undergone; stated that he had taken medicine, had been dieting himself, and believed he would soon be well again; that he had sent for me, not to prescribe, being always his own physician in slight attacks, but to confer with me respecting some of the peculiarities of his constitution, which he thought it important that his physician should be aware of. He mentioned his liability to neuralgia, affecting his head, stomach, and often his extremities; that he had been, early in life, a martyr to dyspepsia; that for the last few years he had avoided these dyspeptic attacks by a system of diet, confining himself principally to animal food; he had been starving himself for a few days past, in consequence of some return of his old dyspepsia; that when sick, he always required a very stimulating practice; that he slept but little, going to bed early, and rising very early; that he attributed his good health during the last few years to that circumstance. I advised that he should avoid all excitement, that he should remain quiet the next morning in bed, and intermit his official business, which he promised to do. At his request, I called in the evening at 8 o'clock, found him in his parlour, with several of his old military friends; he informed me that he felt much better than he had done for some days; that he thought he would have a good night, and be well by the morning: he was cheerful, and joined in the conversation.

*Saturday, March 27th.*—At 1 o'clock, P. M., I was suddenly summoned to visit the President; found him in bed; told me that he had been attacked about an hour and a half previously with a severe chill; that as usual in the morning he had risen at about 4 o'clock, taken a walk around his grounds, and then to the market-house, and returned to breakfast at half past 7 o'clock; that he had been much occupied all the morning with business, and that the chill had attacked him while engaged with his cabinet. I prescribed the ordinary remedies, such as mustard to the stomach, heat to the extremities, additional bed clothing, and warm drinks. The reaction was slight, and perspiration readily induced by a gentle diaphoretic draught, tartar emetic, with the spiritus Mindereri, and diluents.

I visited him at 5 o'clock, P. M.; his condition was much improved; his skin warm and moist; his thirst allayed; he was cheerful, and said he was satisfied he should have a good night, and be well in the morning; his pulse was soft, about seventy-five; complained only of a slight pain over the right eye, which he considered neuralgic; and which he thought from his own experience would subside in a few hours, and therefore declined using any remedy for it. His tongue being slightly furred, and his bowels not having been moved for two days, I ordered to be taken, at bed time, the following:

℞. Mass Hydrarg. gr. x.
Ex. Colocynth. Comp. gr. iij.
M. ft. pil. No. iij.

this being a medicine which he stated always acted kindly. I left him about half past 6, even more comfortable than at 5.

*Sunday, March 28th.*—At 4 o'clock, A. M., I was summoned to visit the President; found that about 12 o'clock at night he had been seized with a violent pain over the right brow, and in his right side, from which he still continued to suffer: the pains were intermittent, equally increased by deep inspiration and motion, but not by pressure; contrary to his expectation he had slept but little during the night,—none, since the onset of the pain; he complained of thirst; his tongue was dry; his mouth clammy; his skin warm and moist; pulse eighty, and soft; occasionally great nausea. He attributed his pain to the want of an operation from his bowels, which were uneasy. I ordered enemata, sinapisms, with warmth to the part affected, and gave him a Seidlitz powder. Half past 8.—More easy, and disposed to sleep; bowels had been gently opened by the enemata. Ten o'clock.—Has had several light naps; expressed himself much relieved from the pain in his side and head; in other respects much the same as when I left him at half past 8. Finding the bowels had not been sufficiently moved by the injection, which were repeated once or twice, and caused small, dark, offensive, fluid evacuations, with a few lumps of indurated fæces, ordered one of the following pills to be given every two hours:

℞. Hydrarg. Chlorid. Mit. gr. xij.
Pulv. Rhei gr. xv.
Camphoræ gr. vj.
M. ft. pil. No. vj.

and left directions that in my absence, should the pain return, cups should be freely applied to the side. Upon visiting the President, I received the following report:—At half past 11 he was very restless; objected to all local applications to his side; applied laudanum to the rectum to remove the unpleasant effects produced by the injection; gave a pill at 12; pain being increased, at his request applied laudanum to the part; slight chilliness about half past 12, requiring warm applications to his extremities; at 1 o'clock, inclined to perspiration; at 2 gave the second pill, soon after which he had a small discharge of black, fœtid water, similar to those produced by the injection early in the morning. At half past 2 I again saw him; his skin was warmer and drier than it had been; pulse somewhat accelerated; his breathing more hurried; tongue and fauces dry; thirst intense; face a little flushed. Upon examination was satisfied that the lower lobe of the right lung was the seat of pneumonia, complicated with congestion of the liver; but that the acute pain was neuralgic. Continued pills; had cups applied over the side affected; Granville's lotion to the spine, and over the brow. He was relieved very much, although the quantity of blood taken by the cups was very small; he felt the effect of its loss, breaking out into a free perspiration, complaining of nausea, and a sense of faintness. It is proper to state that my intention after the examination was to bleed from the arm; but upon witnessing the effect that position had on his pulse, &c., I preferred the cups.

3 *o'clock.*—Applied a blister over the side, and gave twenty drops of laudanum, with one of the pills. At 4, finding him much relieved by the laudanum, and not having yet procured a free evacuation from the bowels, gave him five grains more of calomel, with ten drops of laudanum; this in a few minutes quieted his stomach, relieved his pain, and he fell into a sweet and calm sleep. From the nature of the case, I felt uneasy respecting the result, and asked for a consultation. At the request of the President and his family, I met Dr. F. May, at 6 o'clock, P. M. We agreed entirely as to the character of his disease, and that the present treatment be continued.

29*th.*—7 *o'clock, A. M.* Met Dr. May. The President had an uncomfortable night, being somewhat disturbed in his breathing with a slight dry cough; urinated freely, and passed several small black and fœtid stools; had taken two of the pills, with the addition of three grains of calomel, and on account of his restlessness, three grains of Dover's powder. At this time his pulse was eighty, and soft; skin warm and moist; slight dull pain in his side more permanent; the bowels not having been freely opened, I ordered a small dose of castor oil, with demulcent drinks.

2 *o'clock.*—Met Dr. May. The President had failed to take the oil; had taken small quantities of the demulcents, with mutton broth; had slept quietly occasionally through the day; breathes heavily when lying on his back; has had but little cough; no expectoration; has had one dark fluid evacuation from the bowels, and passed his water several times; small in quantity, and high coloured; says he is not refreshed by his sleep; has had a general warm perspiration; some exacerbation of fever; pulse ninety, and fuller; tongue dry, brown, and pointed; thirst great. Ordered one of the following pills every two hours, with some drink and nourishment.

℞. Hydrarg. Chlorid. Mit. gr. vj.
Pulv. Antimonialis
Pulv. Ipecac. Comp. $\overline{aa}$ gr. xij.
M. ft. pil. No. vj.

8 *P. M.*—Met Dr. May. No new symptom had occurred except the expectoration of pinkish mucus. Ordered continuance of pills, &c. with a large blister over the right hypochondriac, extending to the epigastric region.

30*th.* 7 *A. M.*—Has passed a comfortable night, with the exception of unpleasant dreams; seems better; says he feels better; pulse eighty; tongue more moist; still furred; less thirst; bowels not having been opened for twelve hours, and he complaining of uneasiness from distension, we ordered one of the following pills every three hours till they operated:

℞. Sub. Mur. Hydrarg. gr. xij.
Pulv. Ipecac. gr. iij.
Pulv. Rhei gr. xv.
M. ft. pil. No. iv.

Continuance of nourishment and drink.

2 *P. M.*—Had taken one of the pills, and the half of another; has had no action on the bowels; can lie on his back, or either side, though easiest on his right side; cough and expectoration the same; some exacerbation of fever; pulse eighty-five; tongue dry; more thirst, and complains of uneasiness in his bowels; ordered a continuance of the medicine, &c.

7 *P. M.*—Met Dr. May. Took no more of the pills of calomel and rhubarb; has had several free evacuations from his bowels, which, debilitating him, a.d being likely to continue, ordered of the following pills every two hours *pro re natâ:*

℞. Sub. Mur. Hydrarg. gr. xij.
Pulv. Opii
Pulv. Ipecac. $\overline{aa}$ gr. iij.
Camphoræ gr. vj.
M. ft. pil. No. xij.

with a little weak brandy-toddy, and nourishment; with hot fomentations to the abdomen.

31*st.* 7 *A. M.*—Met Dr. May. Has taken his pills regularly; has had one or two small stools, lighter colour; expectoration and other symptoms much the same; pulse soft, compressible, and intermittent, about eighty; ordered continuance of pills every three hours, with infusion of serpentaria and seneka; drink and nourishment, and a little wine whey.

2 *P. M.*—Met Dr. May. Has been in a fine warm perspiration all the morning, dosing a little, lying on either side; cough the same, with a more copious expectoration of a yellowish viscid mucus, tinged with blood; pulse now ninety, soft and regular; tongue the same; in other respects the same; discontinue pills; continue the serpentaria and seneka, with some drink and nourishment.

7 *P. M.*—Met Dr. May. Has continued to take the serpentaria and seneca; find he has some exacerbation of fever; gave one of the following pills every three hours:

℞. Sub. Mur. Hydrarg.
Pulv. Ipecac.
Pulv. Antim. $\overline{aa}$ gr. xij.
M. ft. pil. No. vj.

and between each dose ℥ss. of spirit of Mindererus, with 1-12th of a grain of tart. antim., till perspiration and sleep are induced; drink, the same.

*April* 1*st*. 7 *A. M.*—Met Dr. May. The pills and spiritus Mindereri were taken till they produced the desired effect, and he had a good night. At this hour his perspiration was too free, though warm; feels debilitated by it; discontinued all medicine for the present, and ordered cordial nourishment and drinks; applied ung. hydrarg. camphorat. over the whole abdomen and blistered surface.

1½ *P. M.*—Complained of feeling relaxed and uncomfortable; took wine whey, cream, &c.; had a small dark green passage, and more consistent; some incoherence; muttering while dozing; picking at the bed-clothes; baring his breast; pulse soft, and compressible. We had applied blisters to inside of the thighs; says his blister on the side feels pleasant; has had frequent discharges from his bowels, with much dryness of the mouth and fauces; tongue dry and brown; took some carrageen at 2; seemed better; pulse eighty, soft; skin warm; mouth not so dry; thirst less; feels blisters; has taken serpentaria, &c. every three hours.

2½ *P. M.*—At my request, the President and his family added Dr. N. W. Worthington, of Georgetown, and Dr. J. C. Hall, of this city, to our consultation; these gentlemen met Dr. May and myself at this hour. After a minute examination and a detail of the history of the case, they perfectly agreed with us, both in our opinion of the character of the case, and in the propriety of the treatment. At this time the situation of the President was as above described; we agreed to continue the serpentaria and seneka infusion, with the addition of a few drops of the aromatic spirits of ammonia to each dose; and at bed time to give five grains of the hydrargyrum cum cretâ, with an anodyne, if necessary.

9 *P. M.*—Has taken the infusion regularly; could be prevailed on to take but one dose of the ammonia; has had one free evacuation from his bowels; about 8 o'clock, was seized with return of pain in the side, which was readily relieved by the application of warm poultices over the blistered surface and Granville's lotion, along the spine; it then attacked him over the right brow; the lotion relieved it instantly, but it returned to the side. The application of the lotion to the brow and spine at the same time removed the pain entirely; afterwards he complained of soreness and cramp in the gastrocnemius muscle, which were removed by frictions; has been all the evening in a fine warm perspiration; cough, expectoration, &c. much the same. The hydrargyrum cum cretâ was given, with twenty drops of laudanum.

10 *P. M.*—Became restless; moans a good deal; skin moist and warm; pulse, &c., the same; has taken a little nourishment. Dr. Hall remained with me to-night; we agreed to give one of the following pills every two hours till composed, viz:

℞. Hydrarg. Chlorid. Mit.
Camphoræ $\overline{aa}$ gr. vj.
Pulv. Opii gr. iij.
M. ft. pil. No. vj.

*April* 2*d*. 10 *A. M.*—Met Drs. May, Worthington, and Hall. Has passed a comfortable night; took his pills; infusion and nourishment when awake. His tongue is dry and brown; thirst great; skin warm and moist; pulse ninety, soft and regular; some cough; expectoration brownish mucus, tinged with blood; says he does not feel as well as he has done, though he makes no particular complaint; we considered him rather worse; we agreed to give him two grains of blue mass every three hours, with the serpentaria and seneka infusion; nourishment, &c., continued.

6 *o'clock*, *P. M.*—Met Drs. May, Worthington, and Hall. Has taken his pills and infusion regularly; had several small brownish watery evacuations from the bowels, which he says weakened him; he had taken some beef tea, weak brandy toddy, &c.; slept an hour or two; during this sleep some incoherent muttering; had taken a few drops of laudanum to quiet the bowels, and relieve griping. We find him as follows: pulse eighty, soft and full; skin warm and moist; tongue broader and softer; agreed to give half a grain of calomel, half a grain of camphor, and a quarter of a grain of opium, every two hours, with some nourishment, &c. &c.

*April* 3*d*. 12 *o'clock*, *M.*—Dr. Alexander, of Baltimore, being added to the consultation, met Drs. May, Worthington, Hall, and myself. Dr. A. concurred entirely in the view which had been taken of the case, and the treatment pursued. During the night, the tongue, pulse, and skin, had remained as usual, and he had had one copious evacuation, which did not weaken him, though he got up to the chair, as he had insisted on doing, throughout his illness. Sleep, accompanied by muttering, and disturbed by a dry hacking cough, which was relieved by a tea-spoonful of the syrups of squill, morphia, and Tolu, in equal quantities. From 2 to 5 A. M. had slept sweetly; from which time to the present had dozed and muttered, but when aroused his mind was perfectly clear; felt relaxed; had taken wine whey, the pills of calomel, camphor, and opium, being continued.

We found an exacerbation of fever denoted by a reddened and heated skin, increased frequency of the pulse; medicine ordered to be discontinued, and cooling drink to be given.

This excited state soon subsiding, we were compelled to resort again to the cordial drinks and nourishment.

Up to this time he had been propped up in bed, and said that he felt much better; encouraging his friends with an expectation of a speedy recovery.

2 *o'clock, P. M.*—Moaned very much and talked in his sleep.

2½.—Was much exhausted by a very large and feculent discharge; took brandy and water and jelly.

4.—More feeble and languid; gave twenty drops of laudanum to check an inclination to another passage.

4½.—Languor increasing; fell rapidly off into a dozing state, from which it was difficult to arouse him; pulse slow, hobbling, and intermittent. Features shrunken and pinched; skin dark and muddy. The stimulants were urged; sinapisms applied to extremities and abdomen; sent for consulting physicians.

5.—Abdomen distended; more incoherence; had a large serous evacuation from bowels, (still insisted upon getting up to the chair,) by which he was much exhausted; gave starch, laudanum, and kino injection; sponged with hot spirits of turpentine.

6.—Met consulting physicians; we consider the case hopeless: pulse sinking; extremities blue and cold: directed camphor and carbonate of ammonia emulsion, with hot brandy toddy, and frictions.

7.—Had several small serous discharges; stimulating treatment continued.

8. All the mortal symptoms increasing; our efforts to sustain him still continued.

8¾.—He uttered these words, as heard by Dr. Worthington and Mr. Samuel D. Naughan, cupper, leecher, &c., "Sir, I wish you to understand the true principles of the government; I wish them carried out, I ask nothing more." Immediately afterwards he fell into a state of total insensibility, Finally, at half past 12 on the morning of the 4th of April, without a groan or a struggle, he ceased to breathe.

We regret to state that our efforts to obtain a post mortem examination were unsuccessful.

From the above report, faithfully condensed from the notes hourly made at the bedside, it will be seen that the disease was not viewed as a case of pure pneumonia; but as this was the most palpable affection, the term pneumonia afforded a succinct and intelligible answer to the innumerable questions as to the nature of the attack. It was in fact one of our ordinary winter fevers of a low grade, of which pneumonic inflammation, hepatic congestion, and subsequently gastro-intestinal irritation were the prominent traits, and, apart from the distinction of its subject, presents but few points of medical interest. No one could be less prepared to resist such an attack than General Harrison. In early life his constitution had been impaired by hardships and exposure, and of late years by dyspepsia and neuralgia; exercise, regular hours, simple diet, and mental quietude had preserved a frame by no means robust, to a good old age. The change which occurred in all his habits, in consequence of his political relations, and the fatigues and anxieties incident to his official duties after his arrival in Washington, tended to interrupt and disturb the repose of body and mind necessary for the healthful operations of his constitution. Every hour was devoted to the reception of company, or the transaction of the most multifarious and important business. Not only his physical and mental energies were strained to the utmost, but his feelings were often subjected to the severest trials. To counteract the injurious influence of such a mode of life, the greatest care and prudence would have scarcely sufficed, and unfortunately the President did not secure to himself the rest necessary to sustain his strength. He had scarcely enjoyed one night of comfortable repose since his inauguration, and even at his meals was not free from the distraction of company. Under these circumstances the fatal result of his disease is not a matter of so much surprise as of regret.

On the second day his situation was pronounced one of danger; but no certain prognosis could be established by the physicians in hourly attendance.

The deep political and personal interest dependant on the life of the President, imposed on his attending physician a fearful responsibility, of which he felt himself painfully mindful. He speedily sought a consultation, and was scarcely ever absent from the house more than one hour together. Dr. Hall remained with him during the last three nights; Doctors Alexander and Worthington were in attendance with Dr. H. and the attending physician the night of his death: Dr. May being absent from indisposition.

